# Exogenous trehalose largely alleviates ionic unbalance, ROS burst, and PCD occurrence induced by high salinity in *Arabidopsis* seedlings

**DOI:** 10.3389/fpls.2014.00570

**Published:** 2014-10-29

**Authors:** Lei Yang, Xiaoju Zhao, Hong Zhu, Matthew Paul, Yuangang Zu, Zhonghua Tang

**Affiliations:** ^1^Shanghai Chenshan Plant Science Research Center, Chinese Academy of Sciences, Shanghai Chenshan Botanical Garden, ShanghaiChina; ^2^College of Life Science, Daqing Normal University, DaqingChina; ^3^Key Laboratory of Plant Ecology, Northeast Forestry University, HarbinChina; ^4^Plant Biology and Crop Science, Rothamsted Research, HarpendenUK

**Keywords:** salt stress, trehalose, *Arabidopsis*, ion homeostasis, redox state

## Abstract

Trehalose (Tre) has been reported to play a critical role in plant response to salinity and the involved mechanisms remain to be investigated in detail. Here, the putative roles of Tre in regulation of ionic balance, cellular redox state, cell death were studied in *Arabidopsis* under high salt condition. Our results found that the salt-induced restrictions on both vegetative and reproductive growth in salt-stressed plants were largely alleviated by exogenous supply with Tre. The microprobe analysis of ionic dynamics in the leaf and stem of florescence highlighted the Tre ability to retain K and K/Na ratio in plant tissues to improve salt tolerance. The flow cytometry assay of cellular levels of reactive oxygen species and programmed cell death displayed that Tre was able to antagonized salt-induced damages in redox state and cell death and sucrose did not play the same role with Tre. By comparing ionic distribution in leaf and inflorescence stem (IS), we found that Tre was able to restrict Na transportation to IS from leaves since that the ratio of Na accumulation in leaves relative to IS was largely improved due to Tre. The marked decrease of Na ion and improved sucrose level in IS might account for the promoted floral growth when Tre was included in the saline solution. At the same time, endogenous soluble sugars and antioxidant enzyme activities in the salt-stressed plants were also elevated by Tre to counteract high salt stress. We concluded that Tre could improve *Arabidopsis* salt resistance with respect to biomass accumulation and floral transition in the means of regulating plant redox state, cell death, and ionic distribution.

## INTRODUCTION

Abiotic stresses can directly or indirectly affect plant growth and high salinity is one of the most severe abiotic stresses (Zhu et al., 1998; Hasegawa et al., 2000). Under saline condition, large amounts of salt accumulates in the plants and rises to toxic levels in the older transpiring leaves, causing ionic unbalance, burst of reactive oxygen species (ROS) and premature senescence (Zhu, 2001; Munns, 2002; Penna, 2003; Miller et al., 2008; Munns and Tester, 2008). Affenzeller et al. (2009) reported that salinity induced programmed cell death (PCD) in a fresh water green algae *Micrasterias denticulata*. It was proposed that changes in the cytosolic K/Na ratio induced by salinity may be crucial for triggering PCD in living cells (Shabala, 2009). Another important cause of damage might be ROS generated by salt stress (Hasegawa et al., 2000; Zhu, 2001; Miller et al., 2008). In contrast to O_2_, these partially reduced or activated derivatives of oxygen species are highly reactive and toxic, and can lead to the oxidative destruction of cells (Mittler et al., 2004).

Plants have evolved complex defense strategies, including regulations of ionic homeostasis, osmolyte accumulations, and ROS scavenging systems to cope with salt-induced adverse effects (Garcia et al., 1997; Zhu et al., 1998; Vranová et al., 2002; Cuin and Shabala, 2007; Shabala, 2009; Yang et al., 2013). At high salinity levels, plants frequently lack the ability to control Na import, resulting in a serious ionic stress effect (Munns and Tester, 2008). In this case, it is more important for plants to retaining defined K level and K/Na ratio in shoot (Rus et al., 2001; Genc et al., 2007; Jiang et al., 2013; Yang et al., 2013). Pharmacological experiments showed that low concentrations of compatible solutes [glycine betaine, proline, mannitol, trehalose (Tre)] significantly reduces OH-induced K eﬄux and retains K/Na ratio in roots (Cuin and Shabala, 2007). Accumulating evidence showed that compatible solutes such as mannitol, fructans, Tre, ononitol, proline also play active roles in mitigating oxidative stress caused by salinity (Garcia et al., 1997; Zhu, 2001; Tang et al., 2011; Chang et al., 2014). These results suggested that osmolytes including sugars, amino acids in plants function as crucial regulators of redox state and ionic balance in response to salinity. In addition, it has been widely accepted that up-regulating the activities of antioxidant enzymes like superoxide dismutase (SOD), peroxidase (POD), or catalase (CAT) are tightly involved in the detoxification of salt-induced ROS and the avoidance of resulting damage under salinity (Mittova et al., 2004; Affenzeller et al., 2009).

Trehalose is a non-reducing disaccharide, found widely in nature (Fernandez et al., 2010). In bacteria, fungi and insects, Tre functions as a storage carbohydrate and protects against a variety of stresses (Schluepmann et al., 2003, 2012; Paul et al., 2008; Lunn et al., 2014). Not only is Tre storage form, but it also acts as a compatible solute in stabilization of biological structures under abiotic stress in bacteria (Arguelles, 2000) and fungi (Wiemken, 1990). Many bacteria and fungi can utilize Tre as the sole extracellular source of carbon and energy as well as synthesize enormous amounts of the disaccharide as compatible solute (Jorge et al., 1997; Fernandez et al., 2010). Besides its function as a storage carbohydrate and transport sugar, Tre plays an important role in stress protection, especially during heat stress and dehydration (Garg et al., 2002; Elbein et al., 2003; Karim et al., 2007; Luo et al., 2008). In the studies of Eleutherio et al. (1993), it is suggested that Tre accumulation was correlated with heat tolerance when yeast cells were faced heat stress, measured as resistance to 55.5∘C. Upon dehydration, the gene cluster for Tre metabolism in Anabaena was rapidly induced, while Tre accumulation was gradually occurred (Higo et al., 2006).

Because of its originally chemical and physical properties, as well as its demonstrated role in stress management in yeast, fungi and bacteria, it is interested whether Tre is involved in plant stress responses (Iordachescu and Imai, 2008; Fernandez et al., 2010). In rice, Tre has been reported to reduce damage caused by salt stress, leading to preservation of root integrity, chlorophyll loss in leaf blades, growth inhibition and moderation of the expression of the osmosis caused by salt stress (Garcia et al., 1997; Garg et al., 2002; Ge et al., 2008). Recently, it has also been suggested that Tre or its precursor Tre-6-phosphate acts as a signaling molecule in higher plants (Elbein et al., 2003; Avonce et al., 2004; Paul et al., 2010). Feeding Tre to higher plants altered carbohydrate metabolism, nitrogen metabolism, and plant defense (Bae et al., 2005). It was reported that starch levels were increased and sucrose levels were reduced in shoots of *Arabidopsis* seedlings grown on agar plates containing Tre (Wingler et al., 2000; Schluepmann et al., 2003). It was demonstrated that elevated Tre accumulation in rice plants also conferred high tolerance to salt stress: this was ascribed to its role in maintaining potassium in shoots and in reducing the sodium accumulation, so preserving the balance of Na and K (Garg et al., 2002). The recent report also indicated that the improved resistance to high salinity in *Catharanthus roseus* induced by exogenous Tre is associated with ionic regulation and osmotic adjustment (Chang et al., 2014). However, the underlying ecological physiological processes that contribute to the improved salt tolerance by Tre have not been clarified. We hypothesize that salt stress significantly induces oxidative stress and ionic stress. Here, we seek to elucidate whether exogenous Tre can relieve oxidative stress and adjust ion homeostasis under salinity compared to sucrose, and whether the tolerance to salinity is better than sucrose.

## MATERIALS AND METHODS

### PLANT MATERIALS AND GROWTH CONDITIONS

Columbia ecotype (Col-0) of *Arabidopsis* was used as wild type. All seeds were surface-sterilized by 70% ethanol solution containing 1% Triton X-100, washed with ethanol and dried under sterile conditions. The sterilized seeds were then placed on agar plates including MS salt, 1% sucrose, pH 5.7, 0.6% Agar. The plates were kept at 4∘C in the dark for 4 days to synchronize germination and then transferred to light at 23∘C with a 16/8 h light/dark regime. These germinated seedlings were used for different experiments.

### TREATMENT EXPERIMENTS

In the experiment, the 10-days-old wild-type Col-0 seedlings were transferred to perlite substrate and irrigated with 1/2 strength Hoagland nutrient solution. Under this normal growth condition, the plants were cultured for 20 days and then incubated in different treatment solutions. These treatments were untreated nutrient solution (CK), application of 150 mM NaCl (ST1) or 250 mM NaCl (ST2) into CK solution. To study the role of Tre in salt response, exogenous Tre at the concentration of 0.5, 1, and 5 mM was supplemented to the ST1 and ST2 solutions, respectively. Then, to compare the possibly distinct role of Tre and Suc, they were respectively added to ST2 solution at the same concentration of 1 mM. According to Munns (2002), salt concentration in nutrient solution was gradually increased to the ultimate salt dose adapted in this study. After 7 days of treatment, these seedlings were harvested for assays of phenotypic changes, ROS and PCD levels, antioxidant enzyme activities and ionic balance in the leaf and stem of florescence [inflorescence stem (IS)].

### PHENOTYPIC ASSAY

The parameters of flowering rate, inflorescence length, shoot fresh weight, and leaf water content of *Arabidopsis* Col-0 seedlings under all treatments were assayed. The flowering rate was calculated as the ratio (%) of flowering seedlings in relative to the total seedlings in each treatment (around 30 seedlings). The shoots of these seedlings were removed, rinsed with deionized water, sopped up and weighed. The shoot fresh weight and inflorescence length were measured. Each treatment was replicated three times, each replicate consisting of five seedlings. The identical rosette leaves were picked, and their weight and dry weight were recorded after 24 h at 60∘C for calculation of tissue water content (%). Each treatment was replicated three times.

### THE QUANTITATIVE MEASUREMENT OF ROS AND PCD OCCURRENCE IN LEAF PROTOPLASTS USING FLOW CYTOMETRY

Protoplasts were isolated according to the report (Halweg et al., 2005) and modified for our specific application. The above-mentioned rosette leaves used to prepare protoplast were placed upside down in a sterile petri-dish containing 10 mL of protoplast solution (0.4 M mannitol, 5 mM Mes and 8 mM CaCl_2_, pH 5.6, 1% of cellulase R-10, 0.25% macerozyme R-10, and 0.03% BSA). The midrib was removed and the leaf blade cut into pieces of 0.5–1 cm^2^. Then the leaves were incubated for 4 h at 23∘C in the dark. Subsequently, this mixture was passed through a sterile stainless steel mesh sieve (mesh size 100 μm). This filtered protoplast suspension was centrifuged for 5 min at low speed (600 rpm, 25∘C). Intact protoplasts were collected from the interphase and transferred into a new tube. Then 10 mL protoplast washing buffer (5 mM MES, 8 mM CaCl_2_, 0.4 M mannitol, pH 5.6) was added and mixed gently followed by a second centrifugation under the same conditions. This washing step was repeated and a small aliquot of the washed protoplasts was used for the estimation of protoplast density in a hemocytometer. The protoplast density was adjusted to 3–5 × 10^5^ mL^-1^ for ROS and PCD assay.

The determination of protoplast ROS dynamics was performed according to the method (Moon et al., 2003). To measure cellular ROS levels, *Arabidopsis* protoplasts were incubated with 50 μM H_2_DCF-DA for 10 min at room temperature. Fluorescence intensity was then measured by flow cytometry (FCM; Partec GmbH, Bioflow, Martinsried, Germany) with excitation and emission setting of 488 and 530 nm. Experiments were repeated at least three times with around 50,000 protoplasts per assay.

The cellular PCD occurrence, defined as percentage of PCD-occurring protoplasts to the normal ones, was performed with FCM. Briefly, the isolated protoplasts were washed twice with protoplast washing buffer, re-suspended cells in 195 μL pre-diluted binding buffer and added 5 μL Annexin V-FITC kit (Bender MedSystems, USA), mixed and incubated for 10 min at room temperature (Solution 1). The solution 1 was resuspended in 190 μL pre-diluted binding buffer, added 10 μL of the 20 μg mL^-1^ propidium iodide (PI) stock solution. After addition of another 300 μL binding buffer, the suspended protoplasts were analyzed by flow cytometer PAS (Partec GmbH, Bioflow, Martinsried, Germany) with 488 nm band-pass excitation and 530 nm band-pass emission. Three independent sets of experiments were performed.

### MEASUREMENT OF ELEMENT DISTRIBUTION IN LEAF AND INFLORESCENCE STEM

The element distribution in plant organs was detected in a method according to Paul et al. (2003) with some modifications. The harvested leaf and IS samples were sliced into small pieces with a length of 2–3 mm at each side and then rapidly frozen in a 2:1 mixture of propane and isopentane pre-cooled with liquid nitrogen, freeze-dried at -45∘C for 3 days and stored at 20∘C in a desiccator over silica gel for determination of element content. The prepared pieces of sample were subjected to microprobe analysis in a Philips EM 420 with the energy dispersive system EDAX DX-4 (EDAX Inc., Mahwah, NJ, USA). The accelerating voltage was 15 kV, the take-off angle 25∘ and the counting time 60 live seconds. Semi-quantitative weight fraction (Wt%) of elements (Na, K, Cl, Mg, P, S, Ca, C, N, and O) was performed, taking into account the calibration coefficients (Cliff-Lorimer factors) of the elements in relative to K. Ten points of analysis per tissue and three repeats per treatment were analyzed by means of X-ray microanalysis.

### DETERMINATION OF ENZYME ACTIVITY

For the detection of antioxidant enzyme activities, 0.5 g leaf tissue was homogenized at 0–4∘C in 5 mL of Tris-HCl buffer (50 mM, pH 7.0) containing 1 mM EDTA-Na_2_ and 1% (w/v) soluble polyvinyl pyrrolidone. Homogenates were centrifuged at 10,000 g for 30 min at 4∘C. The total soluble enzyme activities were measured spectrophotometrically in the supernatant at 25∘C (spectrophotometer model, UV-160A, Shimadzu Corporation, Japan). Protein concentration in the enzyme extracts was determined according to (Lowry et al., 1951) using defatted BSA as a standard.

Superoxide dismutase (E.C. 1.15.1.1) activity was determined by the modified method of (Beauchamp and Fridovich, 1971). The reaction mixture contained 50 mM sodium phosphate buffer (pH 7.3), 13 mM methionine, 75 mM NBT, 0.1 mM EDTA, 4 mM riboflavin and enzyme extract containing 50 μg protein (100 μL). The reaction was started by the addition of riboflavin, and the glass test tubes were shaken and placed under fluorescent lamps. One unit of SOD was defined as the amount of enzyme that produced 50% inhibition of NBT reduction under assay conditions.

Activity of POD (E.C. 1.11.1.7) was assayed according to the report (Rathmell and Sequeira, 1974). The formation of the conjugate product of guaiacol was measured at 460 nm. The reaction mixture contained 1.8 mL of 100 mM sodium phosphate buffer (pH 6.0), 100 μL guaiacol (62 mg dissolved in 10 mL distilled water), 100 μL of 12 mM H_2_O_2_ and 100 μL of leaf extract. The increase in A_436_ was measured as the conjugate was formed using an extinction coefficient of 26.6 mM^-1^ cm^-1^ for the conjugate.

Catalase (E.C. 1.11.1.6) activity was determined according to the report (Patterson et al., 1984). The decomposition of hydrogen peroxide was followed at 240 nm in a quartz cuvette (extinction coefficient of H_2_O_2_ was 0.04 mM^-1^ cm^-1^). The reaction mix consisted of 2.7 mL 0.1 M sodium phosphate buffer (pH 7.0), 100 μL of 300 mM hydrogen peroxide solution and 200 μL plant extract equivalent to 25 μg protein in a final volume of 3 ml.

### SOLUBLE SUGARS IN LEAVES

Soluble sugars were extracted from the intact leaf discs, with 80% (v/v) ethanol at 80∘C, for 20 min according to the method (Yang et al., 2013; Chang et al., 2014). Sucrose and Tre were separated by reversed phase HPLC on an NH_2_, 250 × 4.6 mm column (YMC-Pack Polyamine II) with acetonitrile/water and detected with an Evaporative Light Scattering Detector (Waters 2420, USA). Each treatment was replicated three times.

### STATISTICAL ANALYSIS

Results were subjected to analysis of variance (ANOVA) to determine the significant difference among treatments using SPSS software (SPSS 17.0, SPSS Inc., USA). When ANOVA was performed, Duncan’s honestly significant difference (HSD) *post hoc* tests were conducted. SPSS was also used to calculate the Pearson’s correlation coefficients.

## RESULTS

### Tre PROMOTES VEGETATIVE AND FLORAL GROWTH OF *Arabidopsis* SEEDLINGS UNDER HIGH SALINITY

**FIGURE 1:**
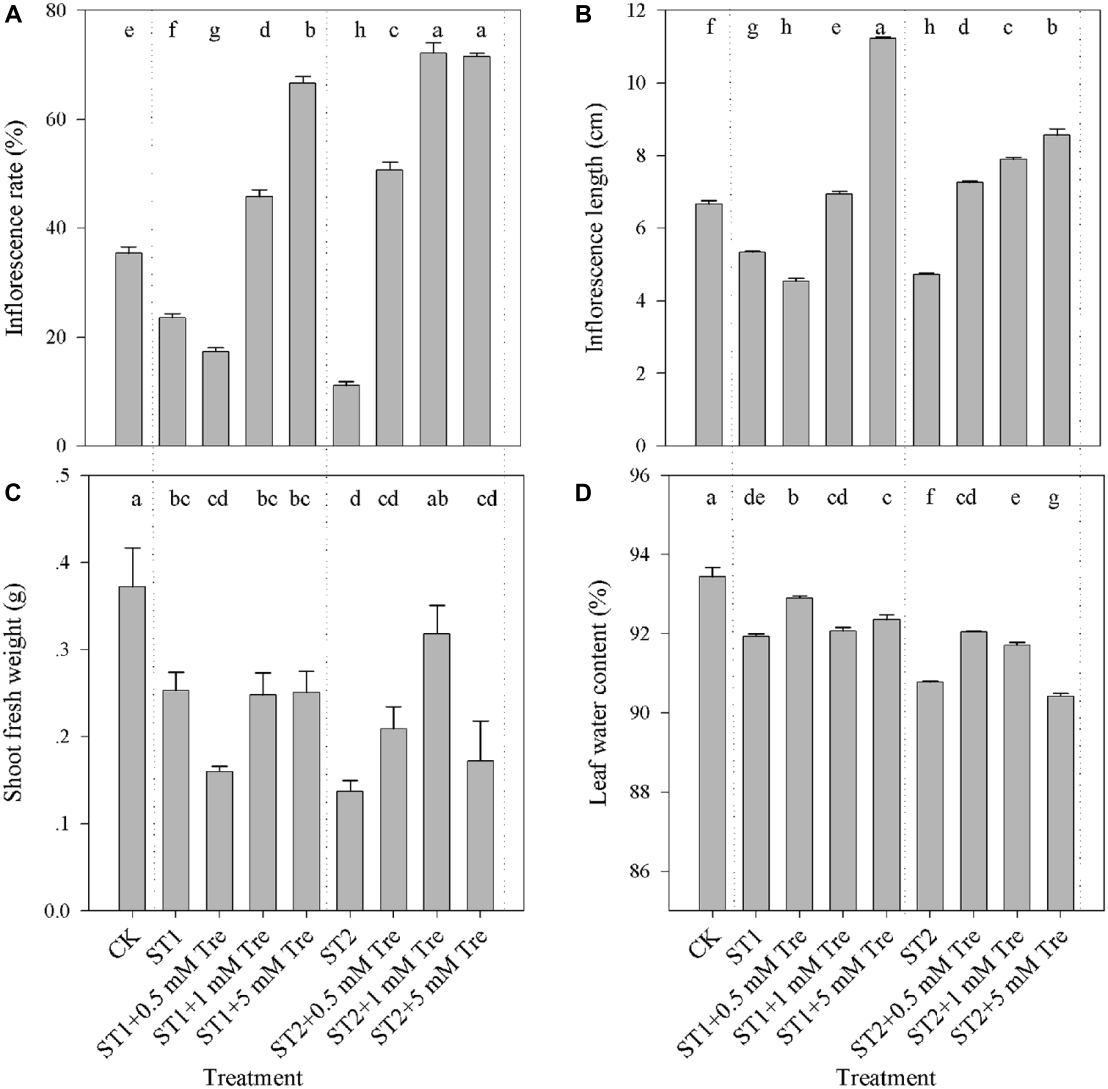
**The vegetative and reproductive growth affected by salinity and Tre in *Arabidopsis*. (A)** Flowering rate (%); **(B)** Inflorescence length; **(C)** Shoot fresh weight; **(D)** Leaf water content. The results showed are the mean ± SE of three replicates and the different letters denote the significant difference among different treatments (*P* < 0.05). ST1, 150 mM NaCl; ST2, 250 mM NaCl; Tre, trehalose.

Salinity is an adverse factor with respect to control vegetative and floral growth of plants. As illustrated in **Figure 1**, the salt-stressed plants with 150 mM (ST1) or 250 mM NaCl (ST2) obviously presented lower flowering rate, shorter IS and less biomass compared with untreated control (CK). The inclusion of Tre over 1 mM in the both saline condition clearly alleviated floral growth inhibition, by virtue of flowering rate and stem length (**Figures 1A,B**). With respect to vegetative growth, we found that Tre exerted a negligible effect to allow plants to recover from the ST1 stress. However, 1 mM Tre generated a remarkably larger biomass accumulation and higher leaf water content in the ST2 + Tre plants than in the ST2 ones (**Figures 1C,D**). These results demonstrated that exogenous Tre could partly rid salt-stressed plants of salt-induced inhibition in biomass accumulation and floral growth.

### HIGH SALINITY AFFECTS ELEMENT DISTRIBUTION IN THE LEAF AND INFLORESCENCE STEM OF *Arabidopsis* SEEDLINGS

**FIGURE 2:**
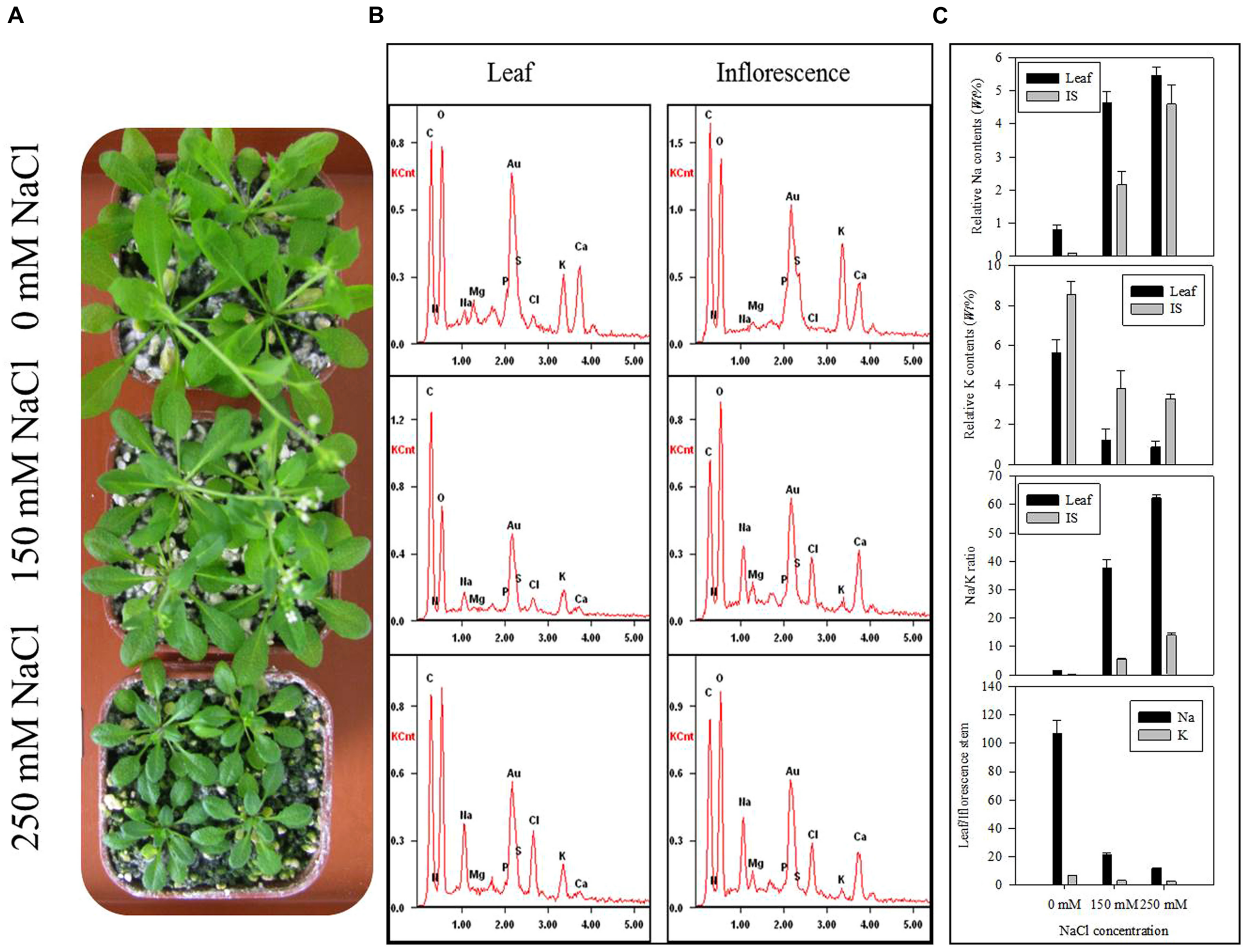
**The ionic accumulations in leaf and IS of seedlings exposed to nutrient solution containing 0, 150, or 250 mM NaCl detected by energy dispersive X-ray spectrometry. (A)** The photographs of control and salt-stressed plants; **(B)** Energy dispersive spectrums generated from energy dispersive system to reflect element distribution; **(C)** Quantitative analysis of ionic contents; IS, inflorescence stem. The results showed are the mean ± SE of three replicates.

To check the distribution of Na, K, and other elements in leaf and IS affected by salinity, we determined them together with other major elements. The seedlings under control or saline conditions displayed obvious difference in morphology and the inhibited leaf area and flowering rate were observed in **Figure 2A**. The energy dispersive spectrums (**Figure 2B**) of element distribution in the control or salt-stressed leaf and IS showed remarkable changes of various ions. With the increased salinity dose, Na contents in leaf or IS both enhanced in a linear way, while K contents were expectedly decreased (**Figure 2B**). Under the ST2 condition, the Na/K ratio had an over 60-fold increase in leaves and 10-fold around increase in IS (**Figure 2C**). The distribution of Na or K in leaf relative to IS was calculated here and there was large difference for Na ratio in leaf/IS, whereas little changes of K ratio in leaf/IS between the control and salt-stressed plants. From the ionic comparison between leaf and IS, we can see that the Na buildup more in the leaves and K accumulated more in IS. The subsequent correlation analysis of Na and K contents in leaf and IS showed that they were mutually correlated in a significant way (*P* < 0.01; **Table 1**).

**Table 1:** Correlations of Na and K contents in the leaf or inflorescence stem from salt-stressed plants.

Correlation coefficient (R)	Na leaf	Na stem	K leaf	K stem
Na leaf	1	0.915**	-0.989**	-0.987**
Na stem	0.915**	1	-0.873**	-0.886**
K leaf	-0.989**	-0.873**	1	0.998**
K stem	-0.987**	-0.886**	0.998**	1

The results showed are the mean ± SE of three replicates and the different letters denote the significant difference among different treatments (*P* < 0.05).

**Correlation is significant at the 0.01 level (2-tailed).

### Tre ALTERS K/Na RATIO AND ELEMENT DISTRIBUTION IN LEAF AND INFLORESCENCE STEM OF *Arabidopsis* UNDER HIGH SALINITY

**FIGURE 3:**
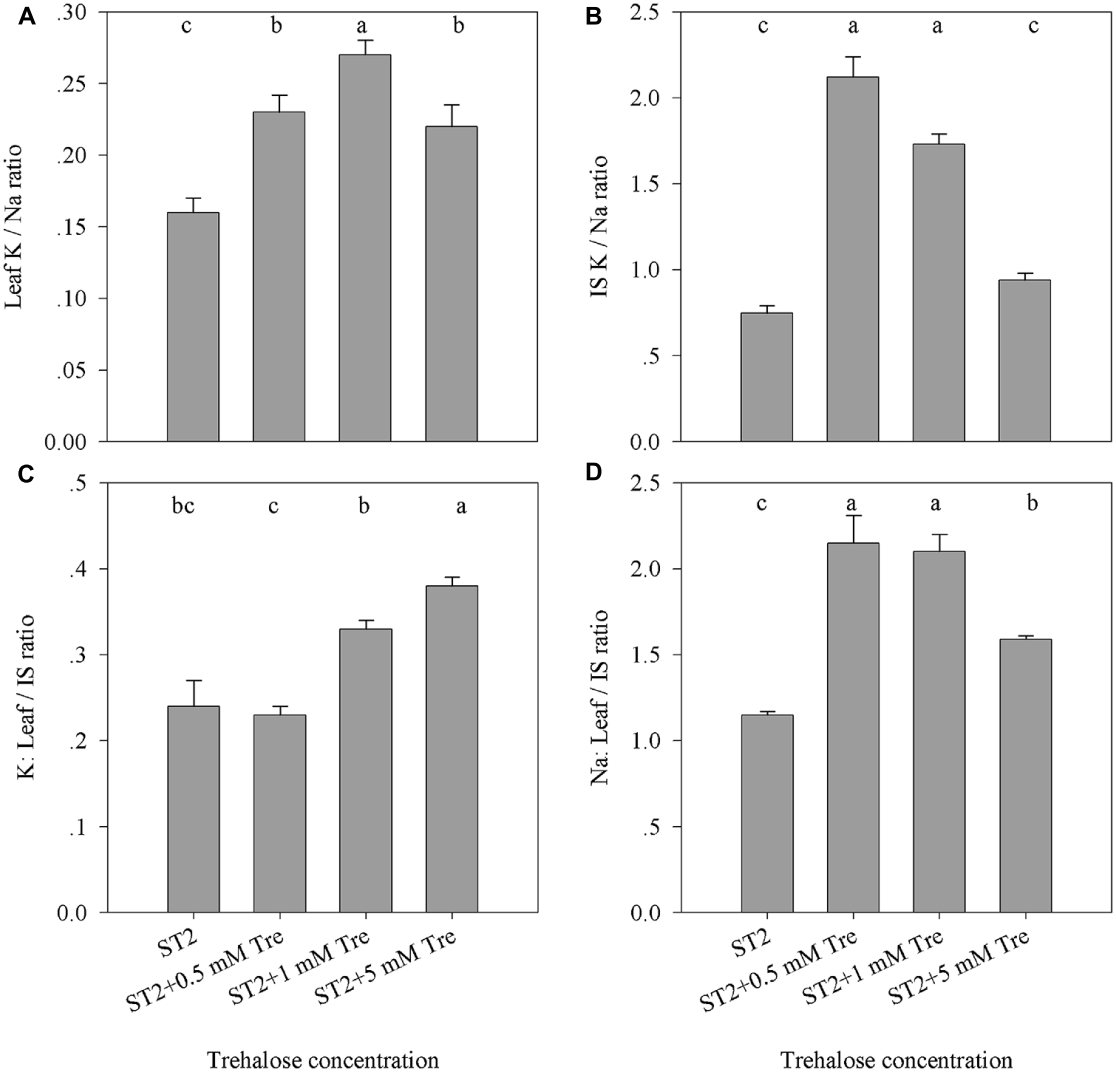
**The effects of exogenous Tre on the ratio of Na and K accumulated in the leaf or IS of plants under ST2 condition. (A)** Leaf K/Na ratio; **(B)** IS K/Na ratio; **(C)** The ratio of K in leaves compared to IS; **(D)** The ratio of Na in leaves compared to IS. ST2, 250 mM NaCl; IS, inflorescence stem. The results showed are the mean ± SE of three replicates and the different letters denote the significant difference among different treatments (*P* < 0.05).

Various elements including Na and K in leaf or IS were then investigated to reveal how they responded to exogenous Tre when plants were incubated in the ST2 conditions. The reason is that our previous results showed that in this salinity situation Tre efficiently improved salt tolerance as to more biomass and successful floral transition. The results showed that leaf Na content was slightly changed, while leaf K was obviously elevated by Tre (**Table 2**). In contrast, exogenous Tre not only largely decreased Na but also promoted K level in IS. These effects of Tre seemed to rely on the concentration used and 1 mM generally had an optimum function in these aspects. As indicated in **Figures 3A,B**, the K/Na ratio was highest both in leaves and IS if with 1 mM Tre in the ST2 situation. In addition, the exogenous Tre affects the distribution of K and Na between leaf and IS. Under the ST2 condition, the distributing ratio between leaf and IS for Na or K was both significantly improved by 1 mM Tre (**Figures 3C,D**). This observation indicated that Tre at a given dose tended to restrict ion flows into inflorescence organs to protect them from salt damages, but this effect might be at the expense of more Na maintained in leaves.

**Table 2:** Element variations in leaf and inflorescence stem (IS) of *Arabidopsis* plants treated with 250 mM NaCl (ST2) in presence of 0 or 1 mM Tre.

Elements (*Wt*)	Leaf		IS	
	ST2	ST2 + 1 mM Tre	ST2	ST2 + 1 mM Tre
Na	53.467 ± 0.644a	51.325 ± 0.663b	46.333 ± 1.033a	24.533 ± 1.419b
K	8.312 ± 0.568b	14.076 ± 0.451a	34.837 ± 2.149b	42.333 ± 1.105a
Cl	35.369 ± 1.616b	54.845 ± 2.515a	49.667 ± 0.913a	28.133 ± 0.956b
Mg	11.597 ± 1.058a	8.392 ± 0.601b	3.787 ± 0.492a	3.967 ± 0.921a
P	7.633 ± 0.751b	12.767 ± 1.444a	8.200 ± 0.781a	5.967 ± 0.491b
S	2.347 ± 0.429b	3.833 ± 0.433a	3.600 ± 0.351a	0.020 ± 0.006b
Ca	61.985 ± 1.286a	44.170 ± 1.097b	6.867 ± 0.296a	7.200 ± 0.458a
C	431.300 ± 3.055a	429.663 ± 1.898a	462.860 ± 3.003b	478.262 ± 2.879a
N	55.900 ± 1.361b	64.823 ± 1.796a	64.893 ± 0.954a	54.157 ± 0.272b
O	337.967 ± 2.614a	334.570 ± 2.643a	324.300 ± 3.213b	364.500 ± 2.434a

*The results showed are the mean ± SE of three replicates and the different letters denote the significant difference among different treatments (*P* < 0.05).*

The exogenous Tre also largely changed the response of other important elements to salt stress. With 1 mM Tre in the ST2 situation, we found that Cl ion was increased in leaves, whereas largely reduced in IS. In this course, the major nutrient elements N, P, and S were observed to be obviously increased in leaves and depressed in IS organ. The elements Mg and Ca were reduced in leaves due to exogenous Tre. The contents of C and O were little changes in leaves and elevated in IS in the ST2 + Tre plants compared to their ST2 controls.

### Tre DECREASES ROS BURST IN *Arabidopsis* UNDER HIGH SALINITY

**FIGURE 4:**
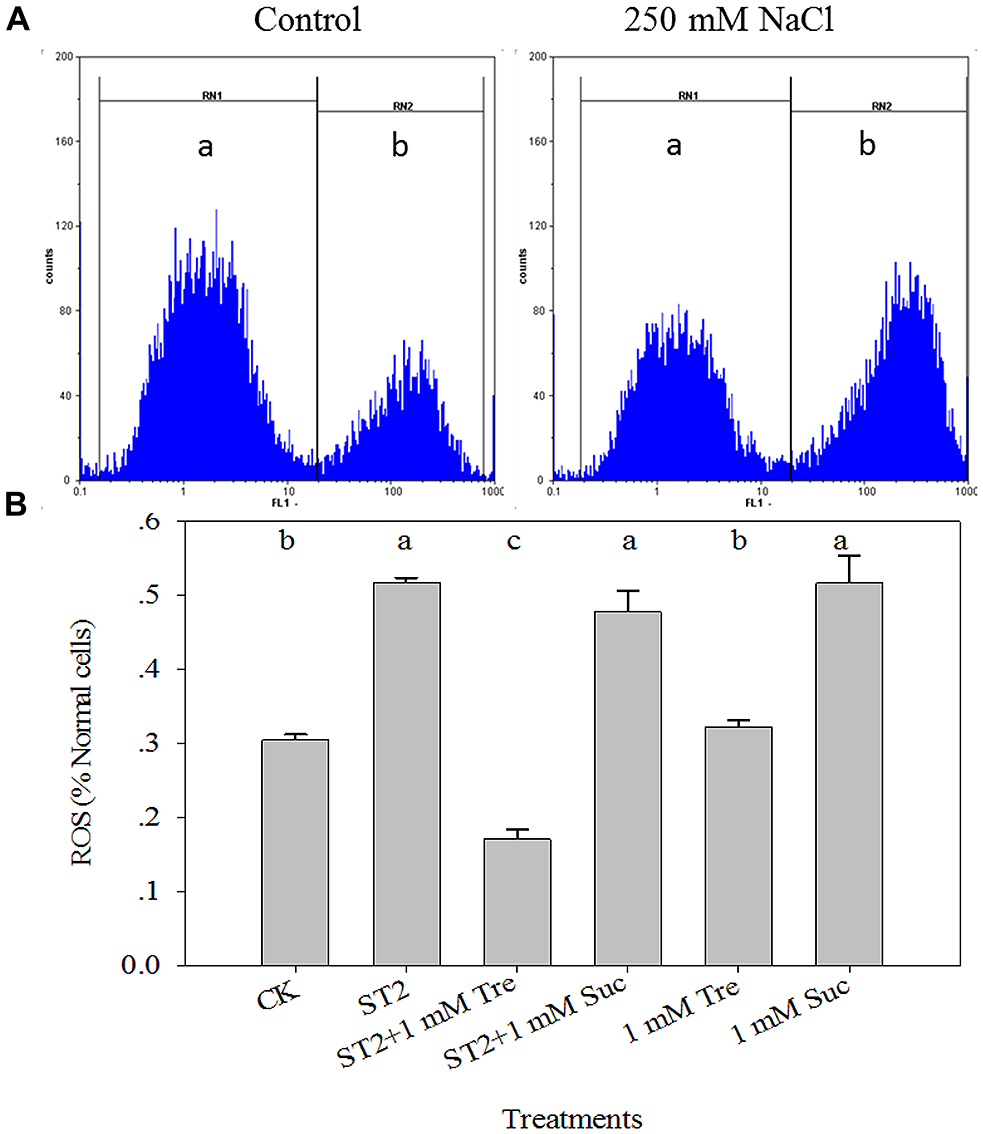
**The leaf ROS dynamics occurring in the protoplasts isolated from plants under control or high salinity conditions. (A)** The schematic illustration of flow cytometry analysis of ROS levels; **(B)** The quantitative results of ROS levels. The protoplasts were stained with H_2_DCF-DA fluorescence solution and analyzed by FCM. The results showed are the mean ± SE of three replicates and the different letters denote the significant difference among different treatments (*P* < 0.05). The zones of (a) and (b) indicate normal and ROS-occurring cells, respectively. ST2, 250 mM NaCl; ROS, reactive oxygen species; Tre, trehalose; Suc, sucrose.

From the above experiments we found that 1 mM Tre presented the best salt tolerance though maintaining K/Na homeostasis, thus we chose 1 mM Tre for the following work. The accumulation of ROS at 250 mM NaCl showed a high level compared with the control. Whereas 1 mM Tre plus 250 mM NaCl (ST2) resulted in fivefold reduction versus 250 mM only (**Figure 4**). In addition, the level of ROS accumulation at 1 mM Tre without NaCl was same as the control (CK) level. This means that only when Tre and salt were be same time, the Tre could be a signal to induce the phenomenon, and improved salt tolerance.

To further substantiate these results, Suc, a non-reducing disaccharide similar to Tre, was used in this study. As shown in **Figure 4**, the accumulation of ROS did not show a significant decrease at 1 mM Suc with ST2, and the treatments with NaCl or Suc resulted in a higher level of ROS accumulation than the control.

### Tre DECREASES PCD OCCURRENCE IN *Arabidopsis* UNDER HIGH SALINITY

**FIGURE 5:**
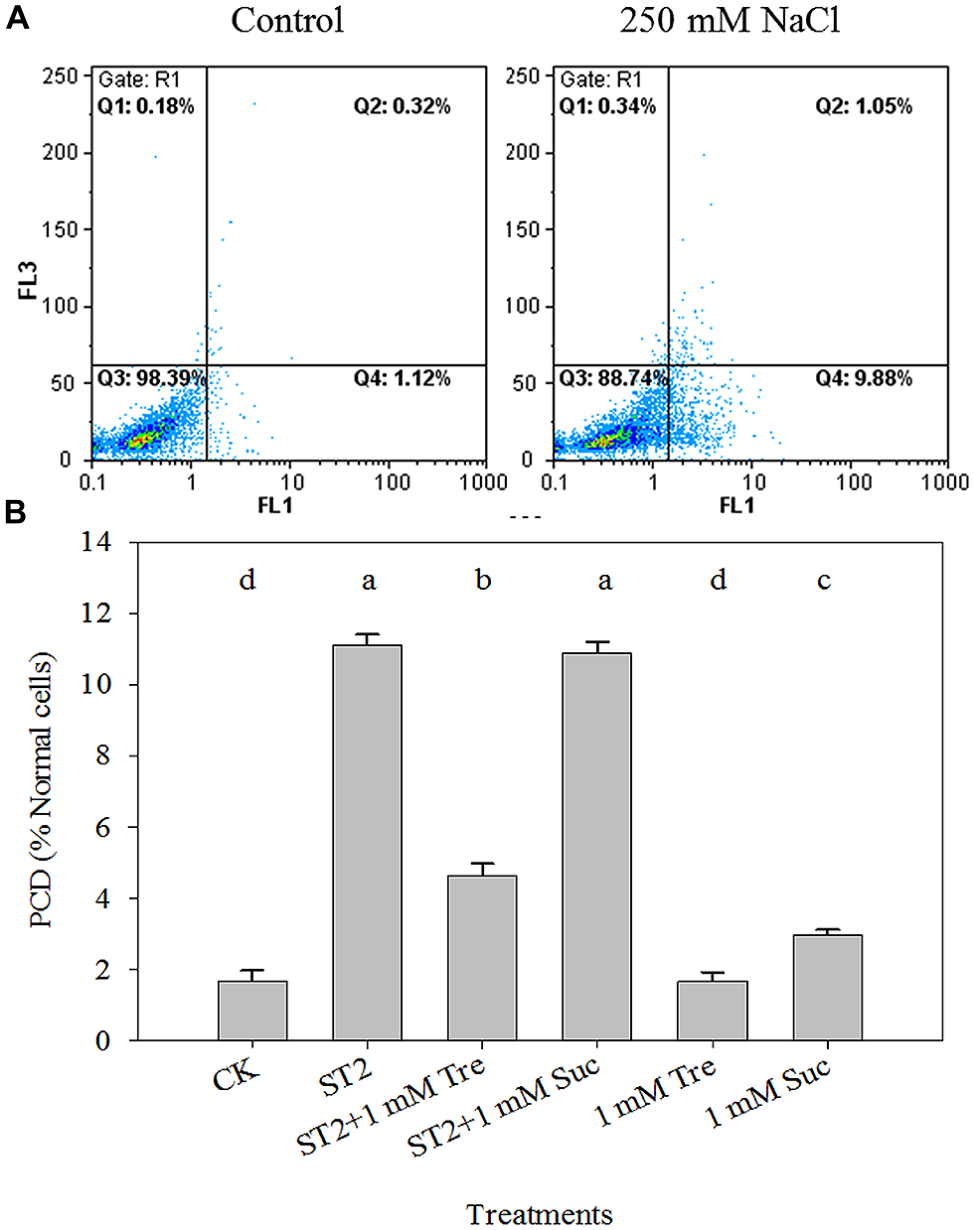
**The leaf PCD dynamics occurring in the protoplasts isolated from plants under control or high salinity conditions and analyzed by flow cytometry (FCM). (A)** The representative FCM diagrams of PCD levels; **(B)** The quantitative results of PCD levels. The protoplasts were stained with Annexin V (FL1 channel) and PI (FL2 channel), and analyzed by FCM. The results showed are the mean ± SE of three replicates and the different letters denote the significant difference among different treatments (*P* < 0.05). ST2, 250 mM NaCl; Tre, trehalose; Suc, sucrose.

Leaf PCD process is tightly related to plant salt response and here it was detected in a quantitative way via FCM method. As illustrated in **Figure 5A**, the percentage of PCD-occurring cells to normal cells obviously advanced when plants were placed in saline condition. The statistic data showed that there is a fivefold increase of PCD level in the ST2 plants than in the untreated controls (**Figure 5B**). The experiment with 1 mM Tre or Suc in the ST2 solution found that PCD occurrence was decreased in the ST2 + Tre plants, while unchanged in the ST2 + Suc ones. This result indicated that Tre could antagonize salinity-induced PCD burst, while Suc did not play this role. Under the condition without salinity, we also found Tre exerted negligible effect on PCD and Suc slightly increased PCD occurrence.

### Tre IMPROVES ENZYME ACTIVITIES OF PART OF ANTIOXIDANTS UNDER HIGH SALINITY

**FIGURE 6:**
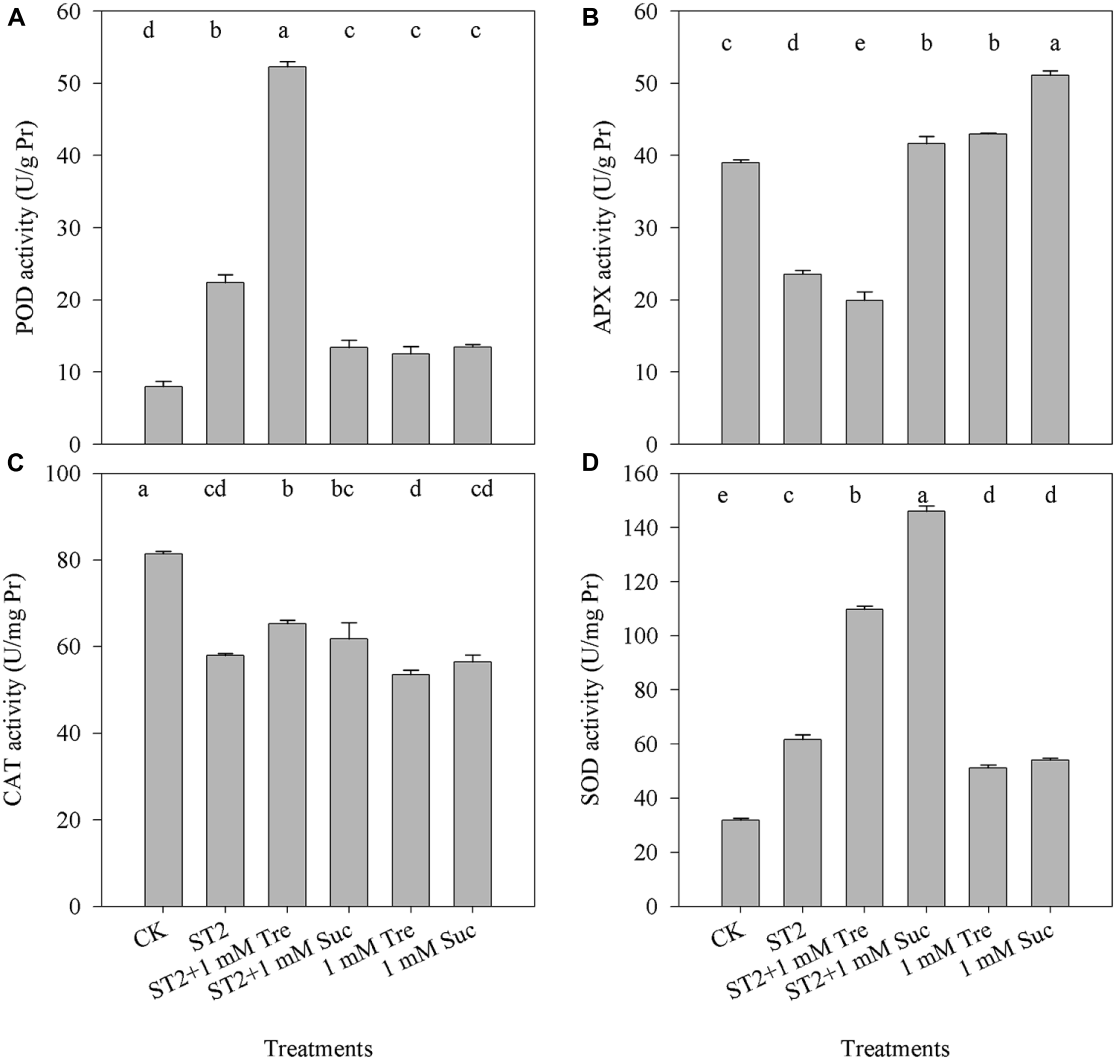
**The changes of leaf antioxidant enzyme activities occurring in the plants under control or high salinity conditions. (A)** POD, peroxidase; **(B)** APX, Ascorbate peroxidase; **(C)** CAT, catalase; **(D)** SOD, superoxide dismutase. The results showed are the mean ± SE of three replicates and the different letters denote the significant difference among different treatments (*P* < 0.05). ST2, 250 mM NaCl; Tre, trehalose; Suc, sucrose.

To understand whether Tre regulates antioxidant system to alter salt-induced ROS or PCD occurrence, we check the activities of four kinds of antioxidants. In reponse to ST2 alone, the activities of POD and SOD were increased, whereas APX and CAT activities decreased than in untreated controls (**Figure 6**). When Tre was included in the ST2 solution, the activity of POD had a twofold increase comparing with the ST2 plants. Other treatments with ST2 + Suc, Tre or Suc only did not obviously altered POD activity. The changes of SOD activity displayed a tendency similar to POD and it was promoted in the ST2 + Tre or ST2 + Suc plants in contrast to the plants treated with ST2 only. Tre included in the ST2 solution did not improved the activity of CAT or APX, while the supplement with Suc to control or ST2 solution both largely promoted APX activities. These results showed that Tre mainly regulated POD and SOD activities to alter plant salt tolerance.

### Tre PROMOTES SOLUBLE SUGAR ACCUMULATIONS IN LEAVES OF *Arabidopsis* SEEDLINGS UNDER HIGH SALINITY

**FIGURE 7:**
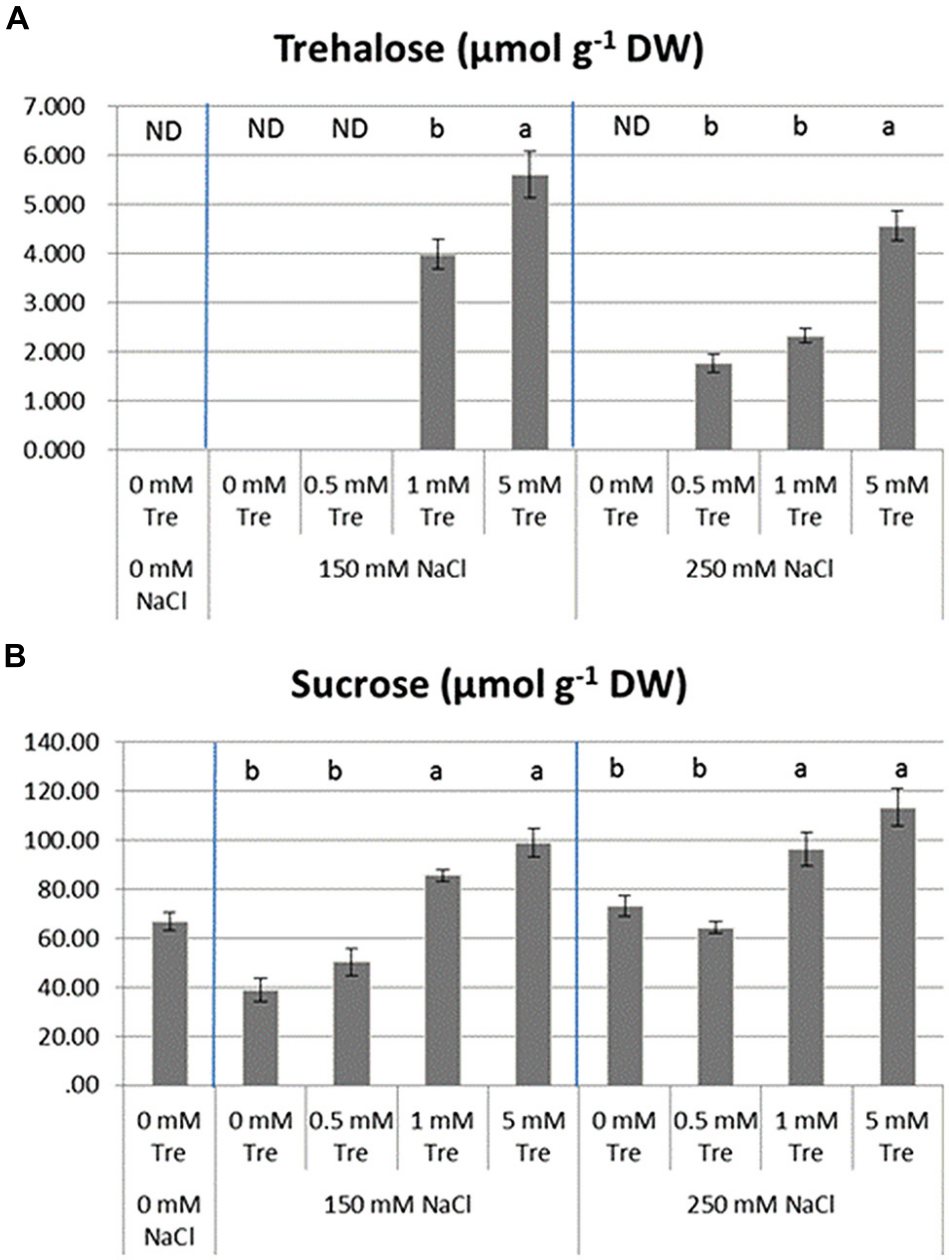
**The effects of exogenous trehalose on leaf soluble sugar levels in the control or salt-stressed plants. (A)** Trehalose. **(B)** Sucrose. The results showed are the mean ± SE of three replicates and the different letters denote the significant difference among different treatments (*P* < 0.05). ND, no detection; Tre, trehalose.

To evaluate how soluble sugars in the stressed seedlings were regulated by exogenous Tre, the internal contents of Suc and Tre in various situations were detected and compared in **Figure 7**. Tre was not found to accumulate in the untreated control and salt-stressed plants (ST1 and ST2). The supplement with various concentrations of Tre to ST1 and ST2 solution induced a detectable level of Tre accumulated in plants to different extent. The concentration at 5 mM of Tre used here achieved an induction of Tre accumulation in a most efficient way both in the ST1 or ST2 plants.

Leaf Suc is normally present in all plants. The treatment with salinity alone did not largely promoted content of Suc and it was significantly reduced in the ST1 plants compared with untreated controls. With Tre included in both salinity situations, Suc was obviously higher than in the plants treated with salinity alone. This inducible effect of external Tre on leaf Suc was more efficient with increased concentration of Tre used.

## DISCUSSION

Accumulating evidence indicates that Tre and its precursor Tre-6-Phosphate (T6P) play important roles not only in metabolic regulation but also in abiotic stress responses in a variety of organisms (Wingler et al., 2000, 2012; Lunn et al., 2006, 2014). These protective properties of Tre are clearly superior to those of other sugars, such as Suc, making Tre an ideal stress protectant (Wingler et al., 2000). However, the functions of Tre in plants still remains a controversial topic, because transgenic plants overexpressing Tre related genes sometimes display growth aberrations and stress symptoms (Fernandez et al., 2010). In higher plants, Tre is not usually present at a detectable level (Paul et al., 2008), and thus its precise role in plants, especially when they are incurring from abiotic stresses, has become an exciting as well as a challenging issue. In this study, we investigated the altered salt responses of *Arabidopsis* modulated by exogenous Tre and the involved mechanisms. During our experiment, the non-salt stress control plants grew well, but salt stress control plants were thin and small. It was observed that about 15 days after high salt treatments, the leaves started to yellow and some seedlings was dead or nearly dead. Because of severe salt damage, the root structure was restricted and nutrient uptake depressed. The addition of Tre to salt stressed plants resulted in a better shoot fresh weight and leaf water content than salt stress control. This phenomenon may be attributed to Tre-induced enzymes involved in the accumulation of storage carbohydrates in photosynthetic tissues (Wingler et al., 2000, 2012). Beside of the benefit of Tre for vegetative growth, floral growth in the salt-stressed plants was also largely improved by the supply of Tre. This observation highlighted the possible role for Tre to help plant to antagonize salt stress in part through completion of life history cycle under high salinity situation. This role of Tre during plant salt response is critical for crop productivity, because severe salt stress will severely interrupt plant floral growth (Munns, 2002; Munns and Tester, 2008). Taking into account vegetative and reproductive growth of the salt-stressed plants affected by Tre, we then addressed the underlying mechanisms of Tre in response to salinity concentrating on ST2 (250 mM NaCl) situation.

Plant growth responds to salinity in two phases: a rapid, osmotic phase that inhibits growth of young leaves, and a slower, ionic phase that accelerates senescence of mature leaves (Munns and Tester, 2008). We observed a notable reduction in leaf area (**Figure 2A**) and water content (**Figure 1D**) after 7 days of ST2 treatment indicating the plants had experienced salinity-induced osmotic stress. The second phase of salt damages to plants is a rapid and temporary increase of Na accumulation and Na/K ratio, especially when plants were exposed to high salinity (Zhu, 2001; Munns, 2002). This will cause the plants to be more susceptible to osmotic and specific ion injury as well as to nutritional disorders that result in reduced yield and quality (Cuin and Shabala, 2007; Shabala, 2009). In our studies, the ionic dynamic of tissue was evaluated by micro-energy dispersive X-ray spectrometry in dry leaves and IS. We found that high salinity used here significantly increased levels of Na and Cl^-^ both in leaves and IS (**Figure 2**; **Table 2**), but decreased K level, suggesting that the impairment in ionic balance had become obvious induced by high salt. We verified the semi-quantitative ionic data in the EDS method in virtue of ICP-MS (inductively coupled plasma mass spectrometry) analysis and obtained the similar results (data not shown). We performed correlations analysis of Na and K contents in the leaf or IS from salt-stressed plants and found that these ions changed in a tight relation (**Table 1**).

It is generally accepted that the maintenance of K/Na homeostasis is an important aspect of salt tolerance (Rus et al., 2001; Cuin and Shabala, 2007). Our results displayed that the K content and K/Na ratio in the leaf and IS were significantly elevated when exogenous Tre included in the saline solution, compared with the salt-stressed control (**Figure 3**). This change of K/Na ratio was more pronounced in the stem of inflorescence than in the leaf due to supplied Tre at optimum concentration of 0.5–1 mM (**Figure 3B**). It was noted that Na accumulation in IS had a 50% reduction in the ST2 + 1 mM Tre plants than their saline controls (**Table 2**). By comparing ionic distribution in leaf and IS, it seemed that Tre was able to restrict Na transportation to IS from leaves since that the ratio of Na accumulation in leaves relative to IS was largely improved due to Tre (**Figure 3D**). The marked decrease of Na ion in IS might account for the promoted floral growth when Tre was included in the saline solution. It was reported that transgenic plants with enhanced Tre levels were able to maintain a higher level of selectivity for K over Na uptake in the roots and were more salt tolerant under the mediate salt condition (Garg et al., 2002). The previous study also showed that treatment with exogenous Tre significantly reduced the salt-induced accumulation of Na in the leaves (Garcia et al., 1997). These results together our observation strongly indicated that Tre metabolism in plants tightly responded salinity by controlling mineral balance.

The FCM measurements of the cellular redox state and cell death in plants have been reported previously and proven to be a quantitative, reliable technology to study ROS and cell death dynamics (Moon et al., 2003; Jiang et al., 2008; Affenzeller et al., 2009; Shabala, 2009). The excess production of salt-induced ROS in plants is a commonly deleterious effect of salinity on plants (Miller et al., 2008). Overproduction of ROS can oxidize plant cell membranes, inducing PCD (Hoeberichts and Woltering, 2003; Mittler et al., 2004). PCD occurrence in plants was tightly linked with lines of biochemical and morphological hallmarks (Hoeberichts and Woltering, 2003). One earlier indicator of PCD before DNA fragmentation is the exposure of phosphatidylserine (PS) on the outer surface of plasma membrane, which can be monitored by FCM with a fluorescent conjugate of Annexin V (Halweg et al., 2005). In virtue of this measurement platform, we showed that high salt stress obviously increased the cellular levels ROS and PCD, whereas exogenous Tre could decrease ROS to release salt tress, and that 1 mM Tre has the most optimum effect (**Figures 4** and **5**). We compared the role of Tre and Suc (sucrose) and found that inclusion of Suc in the saline condition did not decrease salt-induced ROS and PCD occurrence as did Tre (**Figures 4** and **5**). The assay of DNA fragmentation, an apoptotic process, indicated that treatment with Tre suppresses not only water loss but also cell death in the petals, however, treatment with sucrose slightly delayed petal wilting and browning but did not show a significant effect on nuclear fragmentation in petals during senescence (Yamada et al., 2003). Our previous experiment with supplemented sucrose in the nutritional solution showed it will induce increase of endogenous fructose content and fructose/glucose ratio, which is associated with plant senescence process. Considering the sucrose-induced ROS increase in our results, we speculate that the signaling event induced by sucrose and Tre is distinct and sucrose supply to plants might activate fructose signaling cascade, resulting in ROS burst. Accumulating evidence showed plant PCD process during salt acclimation was finely mediated by redox state (Shabala, 2009). The correlation analysis showed the levels of them significantly correlated in our experiment. These results indicated that Tre could alleviate salt-induced cellular ROS and PCD process to modulate plant salt responses.

It was proposed that antioxidant enzyme activity and compatible solutes played important roles in scavenging salt-induced plant ROS burst (Zhu et al., 1998; Hasegawa et al., 2000; Cuin and Shabala, 2007; Harrach et al., 2008). In our studies, high salt stress obviously increased the activities of POD and SOD (**Figure 6**) in response to the higher level ROS under salt stress. While, the activities of APX and CAT were not up-regulated by exogenous Tre, suggesting that Tre is able to scavenges ROS and reduces the salt toxicity mainly by enhancing the activities of SOD and POD. Recent work demonstrated that overexpressing *OsTPS1* enhanced the tolerance of rice seedlings to multiple abiotic stresses by increasing Tre and proline levels and activating some abiotic stress-related genes (Li et al., 2011). Our results showed that endogenous levels of Tre and Suc were greatly up-regulated by exogenous Tre supplied into saline solution (**Figure 7**). The accumulations of some osmotic substances such as proline, glutamate were found to be also largely improved in the salt-stressed *C. roseus* plants when exogenous Tre was supplied (Chang et al., 2014). These results suggested that Tre could modulate plant osmotic substances to cope with salt-induced ROS burst. In addition, the improved sucrose regulated by exogenous Tre likely contributes to promote floral growth inhibited by salinity.

In conclusion, we found that exogenous Tre had considerable potential to release salt-induced restriction on both vegetative and reproductive growth in *Arabidopsis* under high salt condition. The analysis on the ionic homeostasis showed that Tre could help salt-stressed plant retain higher K and K/Na ratio in the leaf and stem of inflorescence. The cellular redox state, cell death, antioxidant system, and soluble sugars were all found to be controlled by Tre to improve salt tolerance. We present here the clear evidence that Tre could improve plant salt tolerance and exogenous Tre likely acts as an elicitor of biological process involved in plant salt stress responses.

## AUTHOR CONTRIBUTIONS

Lei Yang, Xiaoju Zhao and Hong Zhu performed the experiment. Zhonghua Tang designed the study and drafted the work. Matthew Paul helped in data analysis and manuscript preparation. Yuangang Zu revised the manuscript.

## Conflict of Interest Statement

The authors declare that the research was conducted in the absence of any commercial or financial relationships that could be construed as a potential conflict of interest.
